# Chronic kidney disease-related atherosclerosis - proteomic studies of blood plasma

**DOI:** 10.1186/1477-5956-9-25

**Published:** 2011-05-13

**Authors:** Magdalena Luczak, Dorota Formanowicz, Elzbieta Pawliczak, Maria Wanic-Kossowska, Andrzej Wykretowicz, Marek Figlerowicz

**Affiliations:** 1 Institute of Bioorganic Chemistry, Polish Academy of Sciences, Noskowskiego 12/14, 61-704 Poznan, Poland; 2 Department of Clinical Biochemistry, Poznan University of Medical Sciences, Grunwaldzka 6, 60-780 Poznan, Poland; 3 Department of Nephrology, Transplantology and Internal Medicine, Poznan University of Medical Sciences, Przybyszewskiego 49, 60-355 Poznan, Poland; 4 Department of Internal Medicine, Division of Cardiology - Intensive Therapy, Poznan University of Medical Sciences, Przybyszewskiego 49, 60-355 Poznan, Poland; 5 Institute of Computing Science, Poznan University of Technology, Piotrowo 3A, 60-965 Poznan, Poland

## Abstract

**Background:**

Atherosclerosis is considered the major cause of the dramatic increase in cardiovascular mortality among patients suffering from chronic kidney disease (CKD). Although the close connection between atherosclerosis and kidney dysfunction is undeniable, factors enhancing CKD-mediated plaque formation are still not well recognized.

**Results:**

To increase our knowledge of this process we carried out a comparative proteomic analysis of blood plasma proteins isolated from 75 patients in various stages of renal dysfunction (CKD group), 25 patients with advanced cardiovascular disease (CVD group) and 25 healthy volunteers (HV group). The collected samples were subjected to 2D electrophoresis. Then, individual proteins were identified by mass spectrometry. The comparative analysis involving CKD and HV groups showed a differential accumulation of α-1-microglobulin, apolipoprotein A-IV, γ-fibrinogen and haptoglobin in patients with kidney disease. Exactly the same proteins were identified as differentially expressed when proteomes of CVD patients and HV were compared. However, a direct comparison of CKD and CVD groups revealed significant differences in the accumulation of two proteins: α-1-microglobulin and apolipoprotein A-IV.

**Conclusions:**

The obtained results indicate that at least two processes differentially contribute to the plaque formation in CKD- and CVD-mediated atherosclerosis. It seems that the inflammatory process is more intense in CKD patients. On the other hand, the down- and up-regulation of apolipoprotein A-IV in CVD and CKD groups, respectively, suggests that substantial differences exist in the efficacy of cholesterol transport in both groups of patients.

## Background

Atherosclerosis, characterized by the accumulation of lipids, inflammatory cells, and connective tissue within the intima-media layer of the arterial wall [1], is a well documented syndrome associated with cardiovascular disease (CVD). During the last decades the wide spectrum of factors that support the development of atherosclerotic CVD has been determined. This long list includes: age, sex, lipid disturbances, hypoalbuminemia, hyperuricemia, anemia, hyperhomocysteinemia, coagulation anomalies, insulin resistance. Atherosclerosis has also been recognized as one of the most serious and frequent complications occurring in patients suffering from chronic kidney disease (CKD). Interestingly, CVD risk factors mentioned above seem to be less essential or even unessential for the emergence of CKD-related atherosclerosis (CKDA).

The formation of atherosclerotic plaques is considered the major cause of the dramatic increase in cardiovascular mortality among CKD patients. Recently, it has been shown that atherosclerosis can accompany even the early stages of CKD. This means that the plaque appears long before the end-stage renal disease (ESRD) is developed [2]. A number of studies suggest that both the acceleration of atherosclerosis and the increase of the cardiovascular event risk correlate with the reduction of glomerular filtration rate (GFR) [3-5]. Compared to the general population, patients with CKD have a 20-fold higher prevalence of premature arterial atherosclerosis [6].

Considering the earlier observations one can distinguish at least two types of atherosclerosis: the "classic" type associated with CVD and the "non-classic" type associated with CKD. They can produce similar clinical symptoms, but the mechanisms which underlie the formation of CVD- and CKD-related plaques are not necessarily identical. The composition of atherosclerotic lesions in CKD patients and CVD patients with no renal function impairment is different. Histological studies demonstrated that atherosclerotic plaques collected from the CKD patients contain more calcium deposits within their intimal part but the content of collagenous fibers and smooth muscle cells is reduced [6,7]. It seems that the atherosclerotic plaque that forms during chronic kidney disease includes more inorganic substances. However, the question of whether the higher plaque instability observed in CKD patients results from extended calcification remains open [8].

Recently, a number of uremia-associated factors enhancing the progression of atherosclerosis were identified [9]. Some of them are also encountered in the general population, but others appear to be more specially linked to CKD: uremic toxins, hypervolemia, chronic microinflammation, oxidative stress, endothelial dysfunction, intravenous iron therapy and disturbances of the calcium-phosphate metabolism.

Although the close connection between atherosclerosis and kidney dysfunction is undeniable, mechanisms that enhance the formation of plaque in CKD patients are still unclear. To increase our knowledge of this phenomenon we carried out a standard comparative proteomic analysis of blood plasma proteins isolated from three groups of subjects: CKD patients, CVD patients and healthy volunteers. As a result we identified four proteins whose accumulation changes during CKD development. In addition, we found that two of them accumulate differently in CKD and CVD patients.

## Methods

### Subjects and samples

This study involved 125 persons divided into five equal groups. The majority of them were patients with CKD (75 persons; 21 females, 54 males), treated in the Department of Nephrology, Transplantology and Internal Medicine, at Poznan University of Medical Sciences. Based on The National Kidney Foundation Kidney Disease Outcomes Quality Initiative guidelines [10] the examined CKD patients were divided into three groups according to their eGFR, calculated by the MDRD formula [11]. The first group, CKD1-2, included patients in the initial stages of CKD (stages 1^st ^and 2^nd ^of CKD, with eGFR = 90-60 ml/min/1.73m^2^). The second group, CKD3-4, included pre-dialyzed patients (stages 3^rd ^and 4^th ^of CKD, with eGFR = 59-15 ml/min/1.73m^2^). The third group, CKD5, comprised patients with ESRD (stage 5^th ^of CKD with eGFR <15 ml/min/1.73m^2^) hemodialyzed for 39.6 ± 9.5 months (mean ± SD) with prescriptions of 4.5-5.5 h/session, 3 times per week. The fourth group (called CVD) included 25 patients with CVD with a history and symptoms of cardiovascular disease and cardiovascular instability. No subjects from this group had any clinical symptoms of renal dysfunction. The fifth, control group (HV) contained 25 healthy volunteers matched with the studied groups for age, gender, race, physical activity, tobacco use and BMI, with normal renal function, with a negative history of acute or chronic inflammation and without any clinical symptoms of atherosclerosis. Persons with diabetes mellitus, an acute inflammatory process and malignant tumors were excluded from the study. Demographic data and clinical characteristics of the studied population (n = 125) is shown in Table 1.

**Table 1:** Demographic data and clinical characteristics of the study population (n = 125)

	CKD1-2	CKD 3-4	CKD5	CVD	HV
age [years]	60.1 ± 8.01	59.9 ± 8.4	60.1 ± 10.1	59.4 ± 9.08	59.5 ± 11.2

males/females	7/18	7/18	7/18	7/18	7/18

body mass index [kg/m2]	28.19 ± 3.05	26.3 ± 2.01	24.7 ± 3.7	28.3 ± 3	24.6 ± 2.1

eGFR [ml/min/1.73m^2^]	77.04 ± 22.9	20 ± 7.9	5.75 ± 7	92.7 ± 21.1	123.6 ± 17.6

arterial hypertension	25 (100%)	25 (100%)	25 (100%)	25 (100%)	0 (0%)

history of myocardial infarction/stroke	4 (16%)	5 (20%)	14 (56%)	17 (68%)	0 (0%)

statin treatment	15 (60%)	12 (48%)	17 (68%)	23 (92%)	0 (0%)

IACE treatment	21 (84%)	12 (48%)	15 (60%)	19 (76%)	0 (0%)

total cholesterol [mg/dL]	217.06 ± 52.89	182.33 ± 29.69	179.53 ± 48.39	191.64 ± 41.22	188.73 ± 33.04

HDL cholesterol [mg/dL]	56.2 ± 11.79	58.4 ± 7.64	46.89 ± 23.076	43.62 ± 10.16	70.62 ± 6.32

LDL cholesterol [mg/dL]	168.66 ± 53.11	120.06 ± 17.11	105.97 ± 40.75	118.02 ± 31.66	93.96 ± 30.21

triglycerides [mg/dL]	171.15 ± 70.1	116.66 ± 24.74	133.35 ± 50.83	149.96 ± 75.76	120.72 ± 37.11

alanine aminotransferase (ALT) [IU/L]	22.51 ± 12.52	16.75 ± 9.96	16.01 ± 7.81	23.39 ± 18.9	15.23 ± 6.37

aspartate aminotransferase (AST) [IU/L]	19.72 ± 14.11	16.35 ± 8.54	15.29 ± 5.61	18.8 ± 9.73	16.05 ± 7.56

bilirubin [mg/dL]	0.59 ± 0.82	0.76 ± 0.17	0.53 ± 0.22	0.49 ± 0.18	0.67 ± 0.154

glucose [mg/dL]	93.47 ± 9.52	91.85 ± 9.17	86.01 ± 10.51	92.28 ± 9.96	82.23 ± 12.19

high sensitivity C-reactive protein (hsCRP) [mg/L]	0.62 ± 0.36	6.58 ± 4.05	12.32 ± 18.86	5.96 ± 2.68	1.29 ± 1.39

carotid artery intima media thickness (CA-IMT) [mm]	0.72 ± 0.16	0.74 ± 0.11	0.77 ± 0.32	0.74 ± 0.19	0.48 ± 0.20

No (%) or mean value ± SD.

The study protocol conforms to the ethical guidelines of the World Medical Association Declaration of Helsinki. Before the commencement of the project, an appropriate approval from the Bioethical Commission of Karol Marcinkowski University of Medical Sciences was obtained. All participating individuals (i.e. CKD-patients, CVD patients and healthy volunteers) provided signed informed consent for treatment and/or study. For all CKD and CVD patients blood samples were collected when standard monitoring blood tests were performed. In the case of hemodialized patients, blood samples were always drawn before the second hemodialyzis session of the week.

Peripheral blood was collected into a closed monovette system containing EDTA and centrifuged immediately at 1000 g for 15 min. The obtained supernatants were then centrifuged at 16 000 g for 15 min at 4°C and frozen at -80°C. Plasma samples were resuspended in IEF buffer (7 M urea, 2 M thiourea, 2% CHAPS 55 mM DTT and 0,5% v/v IPG buffer). Insoluble material was removed by centrifugation at 16 000 g for 20 min. Protein concentration was estimated using a commercial 2-D Quant kit (GE Healthcare).

### 2-D electrophoresis

24 cm IPG strips (pH 4-7, GE Healthcare) were actively rehydrated overnight in IEF buffer contained plasma samples. Each strip was loaded with the same amount of protein (1 mg). The strips were subjected to IEF on IPGphor III (GE Healthcare) using a ramping voltage (50-8000 V) to final 75 000 Vh. After IEF, IPG strips were incubated for 15 min in an equilibration buffer (6 M urea, 2% w/v SDS, 30% v/v glycerol, 50 mM Tris/HCl pH 8.8) with 1% w/v DTT during the first equilibration step or with 2.5% iodoacetamide w/v during the second equilibration step. Second dimension was performed in 11% polyacrylamide gels using the Ettan DALT six system (GE Healthcare) according to the manufacturer's guidelines. For each sample, a 2D analysis was repeated three times. After electrophoresis, gels were stained with Blue Silver overnight [12] and scanned (with Umax scanner, GE Healthcare) using LabScan program.

The images were analyzed using the Image Master Platinum software version 6.0 (GE Healthcare). Spots were detected automatically without filtering. Gel patterns were automatically matched together between classes. In addition, all individual matched spots were validated manually to ensure the correctness of spot matching. To find differently expressed proteins, gap and ratio measures were taken into account. For the ratio, a threshold greater than 1.4 was selected. The relative abundance of each spot (%vol) was calculated as its volume divided by the total volume of matched spots. Spots showing greatest variations were subjected to a Shapiro-Wilk normality test to check the normal distribution of the analyzed population. Analysis of variance between all classes was performed using the ANOVA test to check significant differences between the % volume of each spot in all classes. Unpaired Student's t-test was performed to compare between two particular classes. *P *values <0.05 were considered statistically significant. All statistical analyses were performed using Statistica ver. 8.0 software.

### Mass spectrometry (MS)

Protein spots were manually excised from gels, transferred to Eppendorf tubes and digested with trypsin. The proteins were identified using MALDI-TOF mass spectrometers. The acquisition of MALDI spectra was performed on an Autoflex MALDI-TOF (Bruker Daltonics, Germany) mass spectrometer operated in reflector mode and using delayed ion extraction. Positively charged ions in the m/z range 820-3500 were analyzed. 0.5 μl of the sample was co-crystallized with CHCA matrix and spotted directly on MALDI AnchorChip target (Bruker Daltonics). For data validation, external calibration was performed with a standard mixture of peptides. Flex control v 2.0 was used for the acquisition of spectra and all further data processing was carried out using Flex analysis v 2.0. Monoisotopic peptide masses were assigned and used for databases research. The proteins were identified by peptide mass fingerprinting using the Mascot (Matrix Science, London, UK) program against MSDB/Swiss-Prot database. The protein search was done using the following search parameters: mass tolerance +/-0.2 Da, one allowed missed cleavage, cysteine treated with iodoacetamide to form carbamidomethyl-cysteine and methionine in the oxidized form.

### Western blot analysis

Western blot analyses were performed according to a standard procedure. Plasma samples were mixed with SB buffer (125mM Tris-HCl pH 6.8, 4% SDS, 20% glycerol, 10% 2-mercaptoethanol) and total protein concentration was estimated using a commercial 2-D Quant kit (GE Healthcare). Samples containing 30 μg of protein were separated by electrophoresis in a 4-12% SDS-polyacrylamide gel (Bis-*Tris *NuPAGE, Invitrogen). The separated proteins were transferred to a PVDF membrane and blocked with PBST (PBS plus 0.1% Tween-20) containing 4% BSA. Blots were then incubated overnight with one of the following primary antibodies (all from Santa Cruz Biotechnology): anti-α-1-m (α-1-Microglobulin (10A12) antibody, mouse monoclonal), anti- apoA-IV (apoA-IV (H-240) antibody, rabbit polyclonal), anti-Hp α (haptoglobin α (B-2) antibody, mouse monoclonal), anti-Hp β (haptoglobin β (H-80) antibody, rabbit polyclonal), anti-Fb α (fibrinogen α (26E7) antibody, mouse monoclonal), anti-Fb β (fibrinogen β (H-270) antibody, rabbit polyclonal), anti-Fb γ (fibrinogen γ (H-194) antibody, rabbit polyclonal). Incubation mixtures contained also anti- α1-antitrypsin antibody (AAT (H-203) rabbit polyclonal antibody) or anti-C9 antibody (C9 (X197), mouse monoclonal antibody) which were used as positive controls. After overnight incubation with primary antibodies, blots were washed and probed with anti-rabbit or anti-mouse IgG secondary antibodies conjugated with Alexa Fluor 633 or 635 (Invitrogen). The images were captured using Fuji FLA-5100 scanner. Semi-quantitative analyses of protein accumulation were carried out with the Image Gauge software version 4.0.

## Results

**Figure 1:**
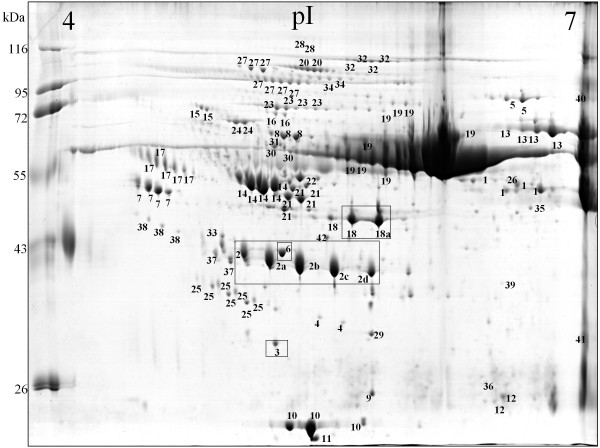
**A representative example of the 2D PAGE analysis of plasma samples collected from CKD patients**. The presented sample was obtained from CKD3-4 patient. IEF was performed in pH 4-7. The proteins identified by mass spectrometry are indexed by numbers (see additional file 1). Differently expressed proteins are boxed.

Earlier it had been demonstrated that the development of atherosclerotic CVD is usually accompanied by significant changes in the accumulation of several plasma proteins [13,14]. Based on this observation we attempted to determine whether similar changes occur during the development of kidney dysfunction-related atherosclerosis. The first question we tried to answer was whether and how the progression of CKD influences the composition of blood plasma proteins. For this purpose plasma samples collected from three groups of CKD patients: CKD1-2 group (25 patients in the initial stage of CKD), CKD3-4 group (25 pre-dialyzed patients) and CKD5 group (25 hemodialyzed patients with ESRD) and from 25 healthy volunteers (HV group) were subjected to 2D electrophoresis. After electrophoresis proteins were visualized, extracted from the gel and examined by mass spectrometry. As a result, 42 proteins were identified out of 160 spots observed in an average gel (see Figure 1 and additional file 1). For all identified proteins the relative levels of their accumulation in each sample were determined (mean from three analyses). Finally, comparative analyses of blood plasma proteomes derived from three groups of CKD patients (CKD1-2, CKD3-4 and CKD5 proteomes) and healthy volunteers (HV proteome) were carried out.

**Figure 2:**
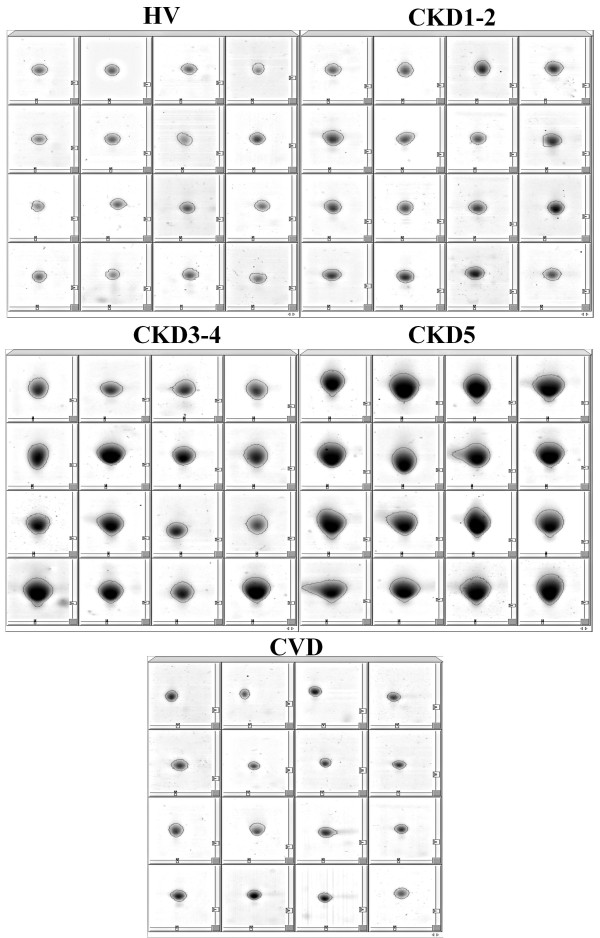
**The close-up sections of α-1-m spots visualized by the 2D PAGE analysis of samples isolated from representative patients belonging to all five examined groups**.

**Figure 3:**
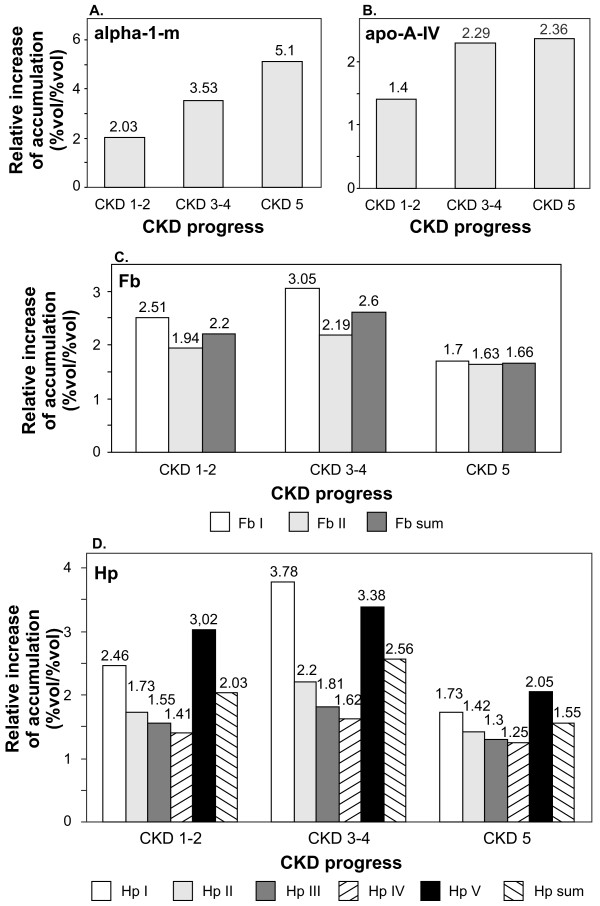
**The relative increase of the accumulation of α-1-m (a), apo-A-IV (b), Fb (c) and Hp (d) in the plasma of CKD patients (calculated against the control group)**. For the actual values of relative abundances, standard deviations, and *p *values see additional file 3.

Proteomes representing the three groups of patients were compared among each other and with the HV proteome. The undertaken analysis revealed that the following four proteins accumulated in the examined samples, at different levels: α-1-microglobulin (α-1-m), apolipoprotein A-IV (apo-A-IV), γ-fibrinogen (Fb), and β-haptoglobin (Hp) (Table 2). For all of them statistically significant differences were observed if any of the CKD groups was compared with the HV group. However, these differences were not always statistically significant when proteomes of CKD patients were confronted with each other (for details see additional file 2). Two out of four identified proteins, i.e.: α-1-m and apo-A-IV occurred only in a single form. The remaining two: Fb and Hp occurred in two and in five isoforms, respectively. The results of 2D electrophoresis suggested that the corresponding isoforms have different isoelectric points (Figure 1). The relative abundance of all four proteins was higher in each group of CKD patients than in the HV group. However, only for α-1-m was a linear correlation between protein concentration and CKD progression observed (Figure 2 and 3a, additional file 3). For apo-A-IV, we found a significant increase of protein accumulation during the initial phase of the disease (in groups CKD1-2 and CKD3-4). Afterwards, it remained at a similarly high level in group CKD5 (Figure 3b). The accumulation of Fb and Hp was increased to the similar levels in all three groups of CKD patients. It is worth mentioning, however, that the concentration of both proteins was usually slightly higher in CKD3-4 than in groups CKD1-2 and CKD5 (Figure 3c,3d). In addition, we found that the concentration of one Fb isoform (FbI) and two Hp isoforms (HpI and HpV) grew more rapidly than the concentrations of corresponding isoforms, especially in groups CKD1-2 and CKD3-4.

**Table 2:** Short characteristic of the proteins differentially expressed in HV and CKD groups as well as in HV and CVD groups

Spot no.	Protein identification	Accession number (MSDB)	Sequence coverage (%)	Queries matched	Score (MALDI)	Mol. mass (kDa)	pI
3	α-1-microglobulin	HCHU	29	8	92	39,8	5,95

6	apolipoprotein A-IV	LPHUA4	52	21	243	45,3	5,23

18,18a	fibrinogen γ-B chain	FGHUGB	39	15	174	52,1	5,37

2,2a,2b,2c,2d	haptoglobin	HPHU2	33	12	155	45,8	6,13

The spots corresponding to these proteins showed at least a 1.4-fold increase or reduction of % of volume.

**Figure 4:**
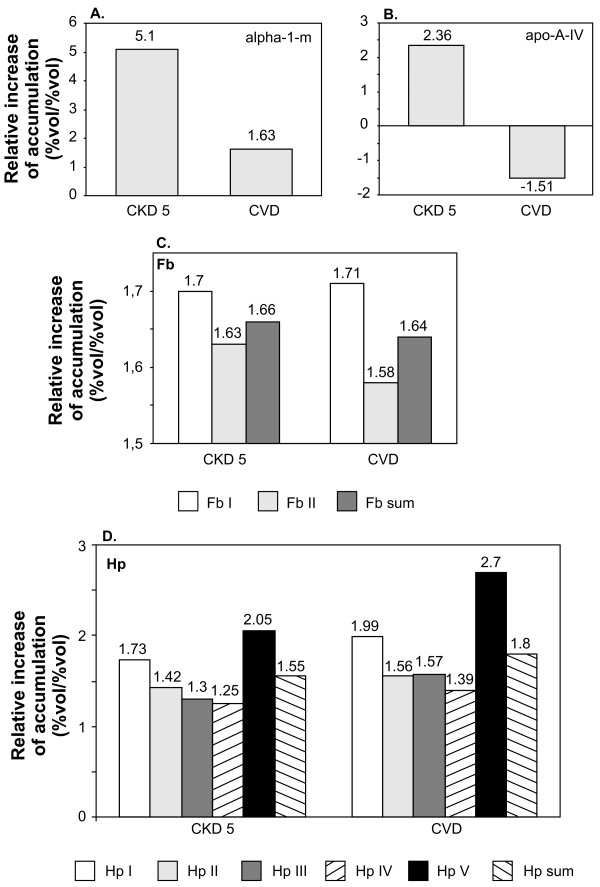
**The relative increase of the accumulation of α-1-m (a), apo-A-IV (b), Hp (c) and Fb (d) in the plasma of patients with advanced symptoms of cardiovascular disease (CKD5 and CVD calculated against the control group)**. For the actual values of relative abundances, standard deviations and *p *values see additional file 4.

Our second question concerned differences between blood plasma proteome in CKD and CVD patients. Because all individuals included to the CVD group displayed the symptoms of the advanced cardiac disease we compared them with the CKD5 group (ESRD patients have much the same symptoms of cardiac disease as patients from group CVD) and with healthy volunteers (group HV was used as a reference). The comparison between CVD and HV groups showed that exactly the same set of proteins (α-1-m, apo-A-IV, Fb and Hp) was differently expressed as earlier when CKD and HV groups were analyzed (Figure 4, additional file 4). Surprisingly, we found that only two proteins, α-1-m and apoA-IV, showed significantly different accumulation in CVD and CKD5 groups. Interestingly, both belong to the set of four proteins differentially accumulating in all three CKD and HV groups and in CVD and HV groups. α-1-m accumulated to higher levels in CVD and CKD5 groups than in the HV group (Figure 4a). However, the increase of protein concentration was significantly higher in the blood plasma of CKD5 patients. A different pattern of changes was observed for apo-A-IV. In comparison to HV, its concentration was increased in the CKD5 group and decreased in the CVD group (Fig.4b). We observed similar profiles of the accumulation of the two remaining previously identified proteins, Fb and Hp, in CVD and CKD5 group (Figure 4c, 4d).

**Figure 5:**
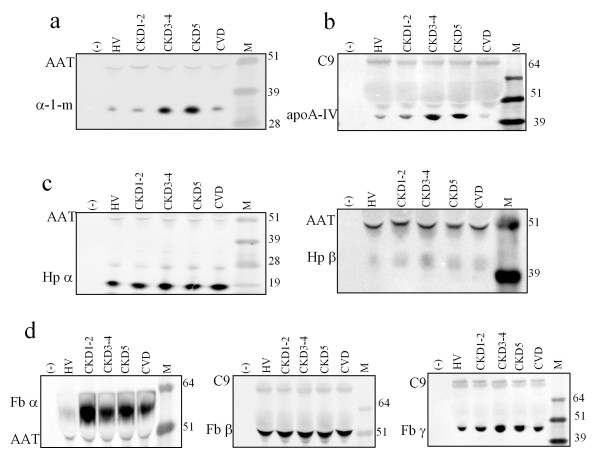
**Western blot analysis of pooled plasma samples (all samples from each examined group were pooled)**. The western blots obtained for: α-1-m (a); apo-A-IV (b); Hp α and β (c); Fb α, β and γ (d). C9 and α1-antitrypsin (AAT) proteins were used as positive controls. (-) - negative control, M-molecular weight marker [kDa].

For verification of our 2DE-MS results, we performed western blot analyses of all four differentially accumulating proteins (α-1-m, apo-A-IV, Fb, and Hp). The analyses included also α-, β-fibrinogen and α-haptoglobin chains which were not found using 2DE-MS method. Western blotting was carried out both for selected individual samples and for pooled samples within each examined group. Based on our 2DE results we decided to use C9 or α1-antitrypsin proteins as positive controls. The representative blots obtained for the pooled samples are shown in Figure 5. Results of their semi-quantitative analysis were consistent with those obtained using a 2DE-MS method. In addition, the data collected revealed that haptogobin α and fibrinogen β accumulated to similar levels in all studied groups. We found some fluctuation in fibrinogen α concentration. However, the differences observed were statistically insignificant.

Because not all patients included to this study were treated with statin or IACE (inhibitor of angiotensin-converting enzyme) we attempted to determine whether the applied therapy affected the accumulation of differentially expressed proteins (α-1-m, apo-A-IV, Fb or Hp). To this end, each examined group of patients (CKD1-2, CKD3-4, CKD5 and CVD) was divided into two subgroups: treated and not treated with statin/IACE. Then for each differentially expressed protein, its accumulation levels in both subgroups were compared. Statistical analysis did not show any significant differences between protein concentrations in corresponding subgroups. *P *values established individually for each protein in all studied groups of patients were always much higher than 0.05 (see additional file 5). This demonstrated that neither statin nor IACE treatment influenced the concentrations of α-1-m, apo-A-IV, Fb, and Hp in patients' blood plasma.

**Figure 6:**
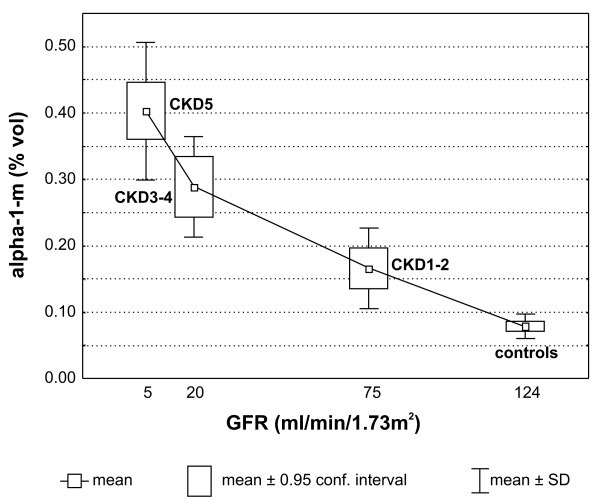
**The inverse correlation between eGFR and the relative abundance (%vol) of α-1-m in the plasma of healthy volunteers and CKD patients**.

At present, eGFR is widely used as one of key parameters characterizing the level of renal dysfunction. According to current standards eGFR above 90 ml/min/1.73m^2 ^is considered a norm. The results presented above suggested that the advancement of kidney disease may also be estimated based on α-1-m protein accumulation in blood plasma. Indeed, we found a correlation between the decrease of eGRF and the increase of α-1-m concentration expressed in %vol (Figure 6). Based on this observation one could expect that α-1-m protein can serve as an early indicator of the impairment of kidney functions.

**Figure 7:**
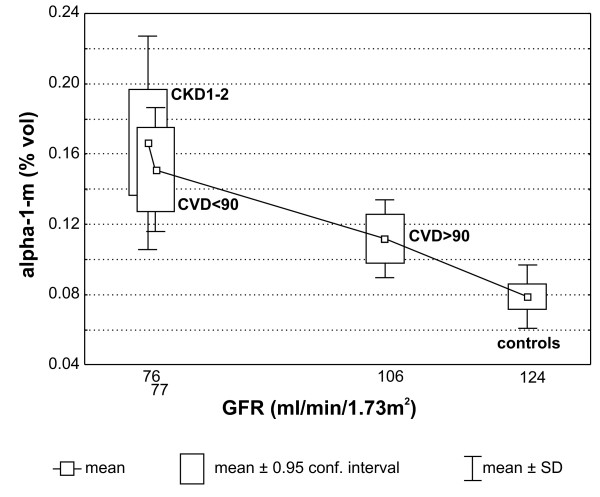
**The inverse correlation between eGFR and the relative abundance (%vol) of α-1-m in the plasma of healthy volunteers, CVD and CKD1-2 patients**.

Because patients suffering from a cardiac disease are at an increased risk of CKD development we attempted to determine whether the first clinical symptoms of renal dysfunction are preceded by the increase of α-1-m accumulation. To this end, we divided CVD patients into two subgroups. The first one, CVD > 90 included individuals with normal eGFR, i.e. 90 ml/min/1.73m^2 ^or higher. The second one, CVD < 90 included individuals with eGFR below 90 ml/min/1.73m^2 ^(mean eGFR in CVD group was 92.6 ml/min/1.73m^2^). Subsequently, we compared α-1-m accumulation in both subgroups and in HV and CKD1-2 group (Figure 7). This revealed that α-1-m occurred at the lowest level in the HV group: the eGFR mean value was 124 ml/min/1.73m^2^. In group CVD > 90 (mean eGFR 106.7 ml/min/1.73m^2^) α-1-m concentration was markedly increased. Such significant increases of α-1-m accumulation were also observed in CVD < 90 and CKD1-2 groups. The mean eGFR calculated for patients classified in these groups was 78.58 and 77.04 ml/min/1.73m^2^, respectively.

## Discussion

The lack of correlation between the well-established risk factors enhancing atherosclerotic CVD and the emergence of CKD-related atherosclerosis [9] clearly indicates that different mechanisms are involved in plaque formation in both diseases. However, the question of how and to what extent these mechanisms differ still remains open. A comparative analysis of blood plasma proteomes isolated from patients in different stages of CKD permitted us to identify four differently expressed proteins: α-1-m, apoA-IV, Fb and Hp. The collected data also suggest that the first two identified proteins i.e.: α-1-m and apoA-IV may find practical application as early markers of nephropathy.

In general, these results seem consistent with observations made by other authors during their studies involving only CKD patients. They have earlier found increased levels of α-1-m in plasma and urine of CKD patients [15-17] and postulated an inverse correlation between eGFR and α-1-m concentration in patients with various renal diseases [18]. There are also a few reports demonstrating the increased levels of apoA-IV in hemodialyzed patients [19,20] and in patients in milder stages of CKD [21]. As a result, a direct correlation between apoA-IV level and CKD progression has already been suggested [22,23].

Elevated concentrations of Hp and Fb proteins in CKD patients' blood or urine have also been reported earlier although little is known about the role of these proteins in CKD development. Both of them are positive acute-phase proteins. Thus, their levels can be increased during inflammation or infection. The increased accumulation of Hp was observed in the urine of patients with nephropathy [24]. The elevated level of Fb was found in the serum of subjects with renal insufficiency [25,26].

Here, we also provide a first direct comparison of blood plasma proteomes isolated from CKD and CVD patients. It demonstrated that the same four proteins (α-1-m, apoA-IV, Fb and Hp) are differently expressed in both groups of subjects (CVD and CKD patients). This would suggest that the differences between mechanisms mediating plaque formation in CVD and CKD are not as significant as we expected. This hypothesis is cast doubt on, however, by the above presented results of our comparative proteomic analysis of CVD and CKD5 patients with similarly advanced symptoms of atherosclerosis. It revealed that two proteins, namely, Hp and Fb are similarly expressed in both groups of patients. However, the levels of α-1-m and apoA-IV accumulation are significantly different. This result indicates that there are some common elements but also some distinct ones in mechanisms of CVD- and CKD-mediated atherosclerosis.

It seems that Fb and Hp proteins play the same role in both diseases. Fb's primary role is to mediate fibrin clot formation and platelet aggregation by binding to cell surface receptors, growth factors, and coagulation factors [27]. In addition, it was shown that Fb binding to thrombin and factor XIII produces clots that are mechanically stiffer and resistant to fibrinolysis [28]. These interactions may explain the association between Fb levels and atherosclerosis. However, they can also contribute to pathophysiological processes including inflammation and thrombosis. The major biological function of Hp is hemoglobin binding. It prevents hemoglobin-mediated renal parenchymal injury and loss of iron following intravascular hemolysis [29]. In addition, haptoglobin can inhibit prostaglandin synthesis and is believed to have anti-inflammatory and antioxidant properties [30]. Thus, higher Hp expression can be most likely classified as a patient's response to atherosclerosis-related stress.

α-1-m is up-regulated in both CVD and CKD patients but its level is more than 3 times higher in the latter group. This may indicate that α-1-m plays the same role in CVD- and CKD-related atherosclerosis although its contribution to both mechanisms is different. Despite extensive studies the function and physiological role of α-1-m protein remain unclear. α-1-m, a member of the lipocalin superfamily, is a low molecular weight protein component of plasma first discovered in pathological human urine [31]. There are several lines of evidence that α-1-m contributes to the regulation of the immune system [reviewed in 32]. Accordingly one can speculate that immunological response is more activated in CKD patients than in CVD patients.

Our most striking finding concerned the apoA-IV expression in CKD and CVD groups. It was up-regulated in subjects with chronic kidney dysfunction and down-regulated in subjects with cardiac disease. ApoA-IV belongs to a wide group of apolipoproteins which participate in lipid transport and metabolism. There are several reports showing that apoA-IV participates in the reverse cholesterol transport pathway [33,34]. It removes cholesterol from peripheral cells and transports it to the liver or to steroidogenic organs where cholesterol is metabolized. Thus one can hypothesize that insufficient synthesis of apoA-IV is one of the important factors affecting atherosclerosis development in CVD patients. On the other hand, in CKD patients reverse cholesterol transport should theoretically be increased due to more effective apoA-IV gene expression. However, the fast progress of atherosclerosis indicates either that other components of this pathway do not function properly or that the production of cholesterol is so high that it cannot be efficiently removed from blood. The data presented here are consistent with earlier observations made by Kronenberg and coworkers. Kronenberg et al. found that low apoA-IV levels are associated with CVD, and this association is independent of triglicerides and HDL cholesterol concentrations. Accordingly, they concluded that apoA-IV may play an antiatherogenic role [35]. Afterwards, the same authors reported elevated concentration of plasma apoA-IV in patients with mild and moderate renal failure [21].

We also found that patient treatment with statin or IACE does not influence the levels of all four differentially expressed proteins accumulation in blood plasma. Moreover, the undertaken comparative proteomic analysis revealed that α-1-m can be used not only as a marker of nephropathy in CKD patients as was postulated earlier [36,37], but also as a predictive marker for selecting among CVD patients with normal eGFR (>90 ml/min/1.73m^2^) those who are in danger of renal failure.

## Conclusions

In general, we have found that the same four proteins differentially accumulate in blood plasma of CKD and CVD patients. Accumulation of these proteins is not affected by treatment with statin or IACE. In case of two proteins: Fb and Hp, the character of the observed changes was similar in both analyzed groups (CKD5 and CVD). However, the remaining two proteins α-1-m and apoA-IV were expressed to significantly different levels. Thus, our results provide an additional line of evidence that different molecular mechanisms are involved in the development of CKD- and CVD-related atherosclerosis. It seems that the former is highly accelerated by the inflammatory processes and to a lesser degree affected by defects in cholesterol transport or metabolism. In addition, we showed that α-1-m protein can serve as an early biomarker enabling the prediction of oncoming renal dysfunction in CVD patients.

## Abbreviations

DTT: dithiothreitol; IPG: immobilized pH gradients; IEF: isoelectric focusing; EDTA: ethylenediaminetetraacetic acid; SDS: sodium dodecyl sulphate; CHCA: α-cyano-4-hydroxycinnamic acid; MALDI-TOF: matrix-assisted laser desorption/ionization-time of flight

## Competing interests

The authors declare that they have no competing interests.

## Authors' contributions

ML conceived, designed and performed the 2DE and MS experiments, participated in data analysis, prepared the first draft of the manuscript.

DF conceived and designed the study, collected the samples for this study, participated in the clinical data analysis and in the first draft preparation.

EP, MWK and AW were involved in collecting samples for this study and clinical data analysis.

MF conceived and designed the study and experiments, participated in data analysis, prepared the manuscript.

All authors read and approved the final manuscript.

## Supplementary Material

Additional file 1 **Proteins identified in the plasma of CKD and CVD patients**. This file provides information about the identified proteins: eg. MSDB/SwissProt accession number, MALDI score and sequence coverage. Spots corresponding to the particular numbers are shown in Figure 1. Click here for file

Additional file 2 **Differences between the relative accumulations of the differentially expressed proteins in all examined groups**. *p *values were generated using unpaired Student's t-test (Statistica ver. 8.0). Differences identified as insignificant (*p *> 0.05) are bolded. Click here for file

Additional file 3 **Relative abundance (%vol) of proteins differentially expressed in HV and CKD groups**. This file provides information about the relative abundance of the proteins differentially expressed in HV and CKD groups, In addition, it presents standard deviations and the results of unpaired Student's t-test generated using the Statistica ver. 8.0 software. Statistically insignificant results are bolded. Click here for file

Additional file 4 **Relative abundance (%vol) of proteins differentially expressed in HV and CKD5 groups as well as in HV and CVD groups**. This file provides information about the relative abundance of proteins differentially expressed in HV and CKD5 groups as well as in HV and CVD groups. In addition, it presents standard deviations and the results of unpaired Student's t-test generated using the Statistica ver. 8.0 software. Statistically insignificant results are bolded. Click here for file

Additional file 5 **Statistical analysis of differences in α-1-m, apo-A-IV, Fb and Hp accumulation in plasma samples derived from patients treated or not treated with statin/IACE**. *P *values were generated using unpaired Student's t-test (Statistica ver. 8.0). This file presents the final outcome of statistical analysis (*p *values) which was undertaken to determine whether the applied treatment affected the accumulation of differentially expressed proteins. *P *values were established individually for each protein in all studied groups of patients. Click here for file
